# Development of an All-in-One Lentiviral Vector System Based on the Original *TetR* for the Easy Generation of Tet-ON Cell Lines

**DOI:** 10.1371/journal.pone.0023734

**Published:** 2011-08-18

**Authors:** Karim Benabdellah, Marién Cobo, Pilar Muñoz, Miguel G. Toscano, Francisco Martin

**Affiliations:** Andalusian Stem Cell Bank, Centro de Investigaciones Biomédicas, Universidad de Granada, Parque Tecnológico Ciencias de la Salud, Granada, Spain; University of Massachusetts Medical School, United States of America

## Abstract

Lentiviral vectors (LVs) are considered one of the most promising vehicles to efficiently deliver genetic information for basic research and gene therapy approaches. Combining LVs with drug-inducible expression systems should allow tight control of transgene expression with minimal side effect on relevant target cells. A new doxycycline-regulated system based on the original *TetR* repressor was developed in 1998 as an alternative to the *TetR-VP16* chimeras (*tTA* and *rtTA*) to avoid secondary effects due to the expression of transactivator domains. However, previously described *TetR*-based systems required cell cloning and/or antibiotic selection of tetracycline-responsive cells in order to achieve good regulation. In the present manuscript we have constructed a dual Tet-ON system based on two lentiviral vectors, one expressing the *TetR* through the spleen focus forming virus (SFFV) promoter (STetR) and a second expressing *eGFP* through the regulatable CMV-TetO promoter (CTetOE). Using these vectors we have demonstrated that the *TetR* repressor, contrary to the reverse transactivator (*rtTA)*, can be expressed in excess to bind and modulate a high number of TetO operons. We have also showed that this dual vector system can generate regulatable bulk cell lines (expressing high levels of *TetR*) that are able to modulate transgene expression either by varying doxycycline concentration and/or by varying the amount of CTetOE vector genomes per cell. Based on these results we have developed a new all-in-one lentiviral vector (CEST) driving the expression of *TetR* through the SFFV promoter and the expression of *eGFP* through the doxycycline-responsive CMV-TetO operon. This vector efficiently produced Tet-ON regulatable immortalized (293T) and primary (human mesenchymal stem cells and human primary fibroblasts) cells. Bulk doxycycline-responsive cell lines express high levels of the transgene with low amount of doxycycline and are phenotypically indistinct from its parental cells.

## Introduction

Inducible gene expression systems based on antibiotics or hormones are a potent research tools and are constantly developed for their use in basic research and/or clinical application. Among the existing inducible transcriptional gene regulatory systems, the reverse Tet transactivator (*rtTA*)-regulatable system is the most widely exploited tool for inducible gene expression. This system, described for the first time by Gossen and Bujard [1] is based on a chimeric transcription factor (the *tTA* transactivator), resulting of the fusion of the bacterial *Tet* repressor (*TetR*) with the activating domain of the herpes virus simplex viral protein 16 (*VP-16*). Random mutagenesis of *tTA* resulted in the *rtTA* (reverse tetracycline controlled *trans*-activator) protein that, contrary to *tTA*, requires the tetracycline to bind the TetO (For review see [2]). The *rtTA*-based system requires the addition of tetracycline to activate transcription (TET-ON system) by allowing the binding of the *rtTA* to the TetO-CMV promoter. Several improvements of the *rtTA* have been done that get better inducibility and reduced background [3], [4], [5], [6]. However, all these tetracycline-inducible systems require a tetracycline-dependant-transactivator to activate the regulated promoter. The requirement of a transactivator for transcriptional activity has several undesired consequences: 1- The regulated promoters are activated by the transactivator and therefore, its natural activity can be altered. 2- Binding of the transactivator to *pseudo-TetO* sites can activate cellular genes. 3- The presence of a transactivating domain makes these proteins very toxic [7], [8], [9], [10]. In fact, several studies have demonstrated that the *rtTA*-based systems can give rise to data misinterpretation due to the toxicity of the transactivator [9], [10], [11].

A new doxycycline-regulated system based on the original *TetR* repressor was developed in 1998, by Yao and colleagues [12]. The original *TetR* do not contain any transactivation domain and rely on blocking the activity of endogenous promoters. These characteristics should allow the design of a less-toxic Tet-inducible expression cassette [12] that maintain the endogenous characteristics of the regulated-promoters and do not transactivate other cellular genes. Using this system, several groups have achieved good results in terms of low leakiness and high induction levels [13], [14], [15], [16], [17]. However, most of these systems are based on two vector systems and are reproducible only if the doxycycline-responsive cells are selected either by cloning [15], [16], [18] or antibiotic selection [17]. One of the potential reasons for this is the high concentrations of *TetR* required to block promoter activity [12], [19]. In spite of the potential advantages of the *TetR over rtTA* to modulate transcription, the development of regulatable vector systems based on *TetR* harbouring all the elements required for doxycycline modulation (all-in-one vectors) has not been explored in detail. Only three all-in-one vectors have been described so far, one system based on herpesvirus simplex (HSV) [19] and two system based on plasmids and/or lentiviral vectors [17], [20]. The use of HSV based vectors has been mainly limited to neural and cancer cells due to their tropism and their toxicity.

Lentiviral vectors are one of the most promising vectors for gene transfer in primary human cells [21], [22]. They are highly efficient and do not express any viral gene that could alter normal cellular physiology. The generation of a highly efficient lentiviral system for easy generation of doxycycline-responsive primary cell lines based on the *TetR* repressor will certainly be of use not only for basic research but also for gene therapy applications [23]. Wiederschain at al described recently a useful all-in-one lentiviral vector able to regulate RNAi. However this system required selection with antibiotics of the doxycycline-responsive cells [17]. In 2001, Ogueta el al. developed an autoregulatable lentiviral vector without the requirement of antibiotic selection [20]. This autoregulatable vector express the *TetR* repressor through an internal ribosomal entry site (IRES) located downstream of the CMVTetO2 promoter. Although this lentiviral system could be of use for several applications, there have been no further publications based on this vector module since 2001. In fact, the same authors observed several potential drawbacks of the system that could limit the use of the vector: 1- The *TetR* repressor and the regulated transgen are expressed through the CMVTetO promoter. Therefore, the steady-state *TetR* concentration that is required to block CMV expression will always allow expression of the transgene. 2- The efficiency of the IRES (from the EMCV) is cell-type specific and this can lead to the lost of doxycycline regulation in important target cells [24].

We have developed a novel two-vector lentiviral system based on the efficient production of two vectors; one expressing the *TetR* repressor through the highly efficient Spleen-Focus-Forming-Virus (SFFV) promoter (STetR vector) and other harbouring the CMVTetO2 doxycycline-regulated expression cassette driving *eGFP*. This dual system allow the easy generation of doxycycline-regulatable cell lines able to express various amounts of transgenes by increasing the multiplicity of infection (MOI) of the regulatable vector. No cloning or antibiotic selection is required to obtain highly responsive cell lines with this system. In addition we have combined all the elements required for Tet-ON regulation into a single lentiviral vector named CEST. CEST contain the doxycycline-responsive cassette (CMVTetO) driving the expression of the transgene (*eGFP*) and the SFFV promoter expressing high amounts of the *TetR* protein. This vector efficiently produced doxycycline-regulated cell lines, including primary human fibroblasts (HFF) and human mesenchymal stem cells (hMSCs).

## Results

### The binary Lentiviral vector system based on the original *TetR* repressor achieves high induction and low background in bulk populations

**Figure 1:**
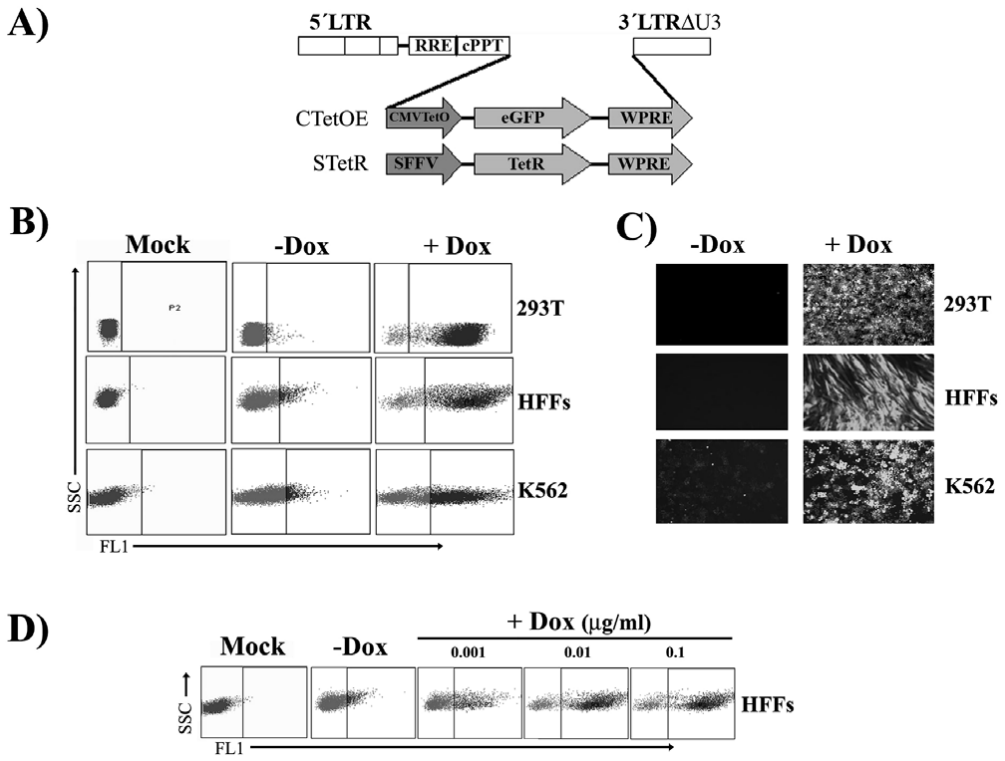
*TetR*-based two-vector system for efficient generation of dose-responsive Tet-On cell lines. **A**) Maps of the two lentiviral vectors required for doxycycline-dependant transgene regulation. The *TetR* repressor is expressed through the constitutive SFFV promoter, highly active in most cell types, including hematopoietic cells. The second lentiviral vector contain the doxycycline-responsive CMV-TetO promoter(Yao et al. 1998) driving the expression of *eGFP*. 293T, primary human fibroblasts (HFFs) and K562 cells were co-transduced with STetR and CTetOE lentiviral vectors at high MOI and analyzed by fluorescence microscopy **(B)** or flow cytometry **(C)** in the absence (–Dox) or presence (+Dox) of 10 ng/ml of doxycycline. **D)** Doxycycline responsiveness (0.001 µg/ml, 0.01 µg/ml and 0.1 µg/ml as indicated) of the Tet-ON HFF cell line derived by cotransduction with STetR and CTetOE LVs.

**Figure 2:**
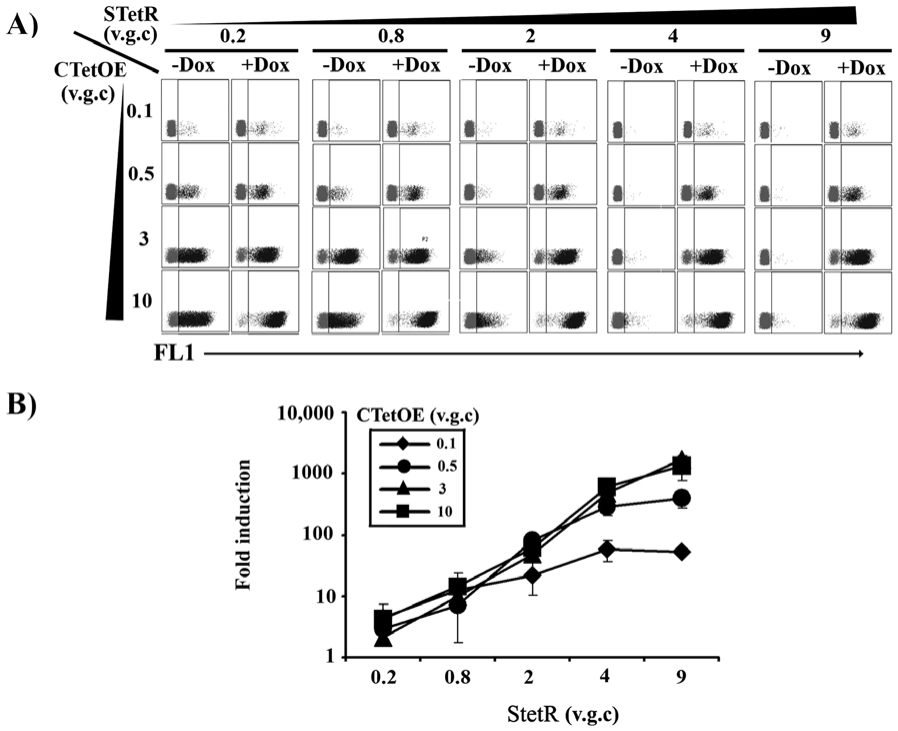
GFP induction level using binary lentiviral vectors. **A**) 293T cells were stably transduced with increasing amount of STetR lentiviral vector. Different *TetR*-expressing 293 T cell lines were generated each harbouring an average of 0.2, 0.8, 2, 4 and 9 vector genomes per cell (v.g.c.) (indicated on the top of Figure A). Each of these *TetR*-expressing 293 T cell lines where later transduced with increasing amount of CTetOE vectors (average of 0.5, 3, 6 and 10 v.g.c.)(indicated on the left of Figure A). Plots show *eGFP* expression of the different cell lines in the absence (-Dox) or presence (+Dox) of 1 µg of doxycycline. **B)**. Graph showing the increment in fold induction of transduced 293 T cells after the addition of 100 ng/ml of doxycycline. The highest induction levels are achieved when the cells contain multiple copies of both StetR (7-9 v.g.c.) and CTetOE vectors (6–10 v.g.c.).

In order to develop a Tet-ON lentiviral system without transactivator domains (such as those based on tTA and rtTA) we used the two expression vectors of the T-Rex Expression System from Invitrogen (pcDNA4/TO-E and pcDNA/TR, Invitrogen). We first constructed an alternative two lentiviral vector system based on pHRSIN-WPRE lentiviral vector [25] (Figure 1A). The first vector, STetR drives the expression of the original *TetR* repressor through the spleen focus forming virus (SFFV) promoter and the second vector, CTetOE, express *eGFP* through the regulatable CMV-TetO promoter (see M&M). The titre (number of transducing units (tu) per millilitre(ml)) obtained with both vectors was above 10.000.000 tu/ml before concentration (data not shown). We used these vectors to generate doxycycline-regulatable 293 T, K562 and primary human fibroblasts (HFF) cell lines. We first transduced the different cell lines with the STetR vector at a MOI of 10 and 7 days later with the CTetOE vectors at a MOI of 2. Seven days later, the different cell lines were incubated in the presence or absence of doxycycline for 48 hours and analyzed for *eGFP* expression by flow cytometry (Figure 1B) and fluorescence microscopy (Figure 1C). All cells kept *eGFP* expression tightly controlled in the absence of doxycycline although with significant differences in terms of regulation efficacy. Tet-ON 293T and HFF cells presented the tightest repression of *eGFP* expression in the absence of doxycycline while Tet-ON K562 cells showed the higher leakiness (*eGFP* expression levels in the absence of doxycycline)(Figure 2C).

We next studied the responsiveness of our dual Tet-ON system to different doxycycline concentrations. Tet-ON HFFs were incubated with increasing doses of doxycycline, from 0.001 µg/ml to 0.1 µg/ml, to determine the minimal dose required for maximal induction. As can be observed in Figure 2, the addition of 0.01 µg/ml of doxycycline was enough to achieved maximal expression, although 0.001 µg/ml already increased *eGFP* expression significantly (Figure 2D). These results showed that the high titre of the STeR lentiviral vector allowed the easy generation of stable bulk cell lines, including primary cells, which are highly responsive to doxycycline after transduction with the regulatable lentiviral vector CTetOE.

### High induction and low leakiness of the *TetR*-based system is dependent on high *TetR* concentration but independent on CMVTetO target sites

Transactivator-containing repressor (*tTA* and *rtTA*) are quite toxic for most cells and must be kept at low concentration. In order to achieve good regulation, the concentration of TetO binding sites must be kept low to equal low transactivator concentrations[26]. We therefore studied whether this was also the case for the unmodified *TetR*-based systems by using STetR and CTetOE lentiviral vectors. We used increasing amounts of STetR to obtain 293 T cell lines with increasing amounts of *TetR* repressor (Figure 2A; from left to right). All these 293T-*TetR^+^* cell lines were transduced with increasing amounts of the CTetOE to obtain increasing concentrations of CMVTetO targets (Figure 2A: from top to bottom). We showed that only those cells expressing high levels of *TetR* repressor have good induction and low leakiness (Figure 2A right panels). A minimum of 2 vector genomes per cell (v.g.c.) of the STetR are required in order to achieve good regulation and over 3–4 v.g.c. give minimal background and maximal induction (Figure 2A; second right graphs). Interestingly, there was a direct correlation between the CTetOE v.g.c. and the fold induction index (Figure 2B). We reached around 1000 fold induction in bulk populations containing 4–9 v.g.c. of the STetR vector and 10 v.g.c. of the CTetOE vector (Figure 2A and 2B) This indicates that the *TetR* repressor, contrary to the *rtTA* transactivator, can be expressed in excess to modulate a high number of TetO operons. This dual vector system can therefore generate regulatable cell lines expressing different transgene amounts by incubating the *TetR^+^* cells with different amounts of the regulatable vector (CTetOE) (see Figure 2A right plots, top to bottom). Each of the different cell lines generated can additionally be modulated by adjusting doxycycline concentrations (as can be observed in Figure 1D for HFF cells).

### Development of *TetR*-based all-in-one LV that efficiently regulates transgene expression

**Figure 3:**
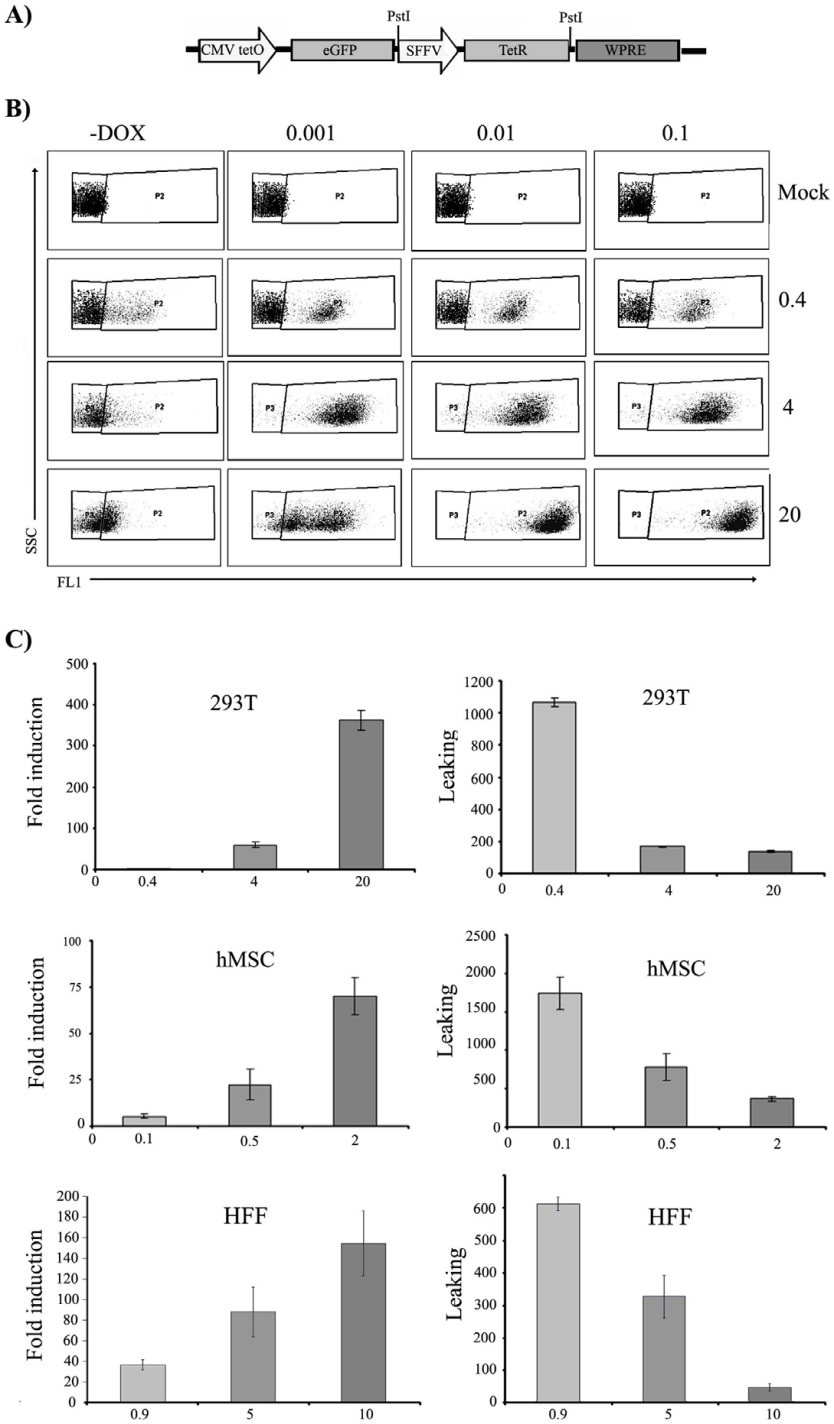
Easy generation of highly responsive cell lines with the all-in-one doxycline-controlable lentiviral vector CEST. **A**). Schematic representation of the CEST. *eGFP* transgene is expressed from a Tetracycline –responsive CMV-TetO promoter and the *TetR* repressor is expressed from the SFFV promoter. **B**) Doxycycline responsiveness of 293 T containing different amounts of CEST vector copy per cell (0 (Mock), 0.4, 4 and 20 v.g.c. as indicated on the left hand side). The different cell lines were incubated in the absence of doxycycline (-Dox), and with 0.001 µg/ml, 0.01 µg /ml and 0.1 µg /ml as indicated on the top. The leaking in the absence of doxycycline decrease as the vector copy number increase (left graphs from top to bottom) **C**) Fold induction (left panels) and leaking (right panels) in 293 T (top panels) and human mesenchymal stem cells (hMSC) (bottom panels) transduced with increasing MOIs of the CEST vector. The average CEST v.g.c. of the different cell lines analyzed are indicated at the bottom of the graphs. The best regulation in terms of higher inducibility and lower leaking is achieved in the cells that contain the highest number of CEST vector integrated.

Although a two vector system has several applications in basic research, single vectors able to deliver the *TetR* repressor and the regulatable expression cassette (i.e. CMVTetO) are highly desirable for the easy generation of doxycycline-responsive primary cell lines and for gene therapy strategies. We therefore constructed an all-in-one dual-promoter lentiviral vector named CEST containing both, the doxycycline-responsive cassette (CMVTetO) expressing *eGFP* transgene and the *TetR* expressing casset (SFFV-TetR)(Figure 3A). We tested the CEST vector in immortalized cells (293 T) and primary cells (hMSCs and HFF). We incubated the different cell lines with increasing MOIs of the CEST vector. Two weeks later, the different cell lines harbouring increasing amounts of the CEST vector were analysed for responsiveness to doxycycline (Figure 3B, only shows for 293 T cells), leakiness (Figure 3C right graphs) and fold induction index (Figure 3C, left graphs). Tet-On cells responded to 0.001 µg/ml in cells harbouring 1–4 v.g.c. of CEST and to 0.01 µg/ml in cells harbouring 10–20 v.g.c. of CEST (Figure 3B and data not shown). Interestingly, as occur for the two-vector system, CEST-Tet-ON cells also requires also several copies of the CEST vector (four or more v.g.c.) to achieve good regulation (Figure 3B and 3C). To analyse this results in detail, leakiness and fold induction index of the different CEST-Tet-ON cell lines were determined and plotted in function of the amount of integrated CEST vector per cell. In all cells analysed we found the same results: cells harbouring the highest amounts of CEST vectors integrated presented the lowest leakiness (Figure 3C right graphs) and the highest fold induction index (Figure 3C, left graphs). We reached up to 400, 180 and 100 fold induction in 293 T (with 20 v.g.c.), HFF (with 10 v.g.c.) and hMSCs (with 2 v.g.c.) respectively. These data corroborate the hypothesis that high concentrations of the *TetR* repressor is the main factor required to get good doxycycline regulation and not the number of TetO target sites. Increased numbers of TetO targets (up to 20 times in 293 T cells; Figure 2B and 2C top graphs) do not preclude *TetR* repression but it does augment transgene expression and therefore increase total fold induction. This property allowed us to control transgene expression not only by adjusting doxycycline concentrations but also by controlling the MOI used for transduction of the target cells (Figure 3B and 3C). The highest transgene expression and the highest inducibility were always obtained with the highest MOIs. CEST-transduced-293T, -HEF and -hMSC expression levels go from a MFI of 179, 260 and 872 in the absence of doxycycline to a MFI of 47956, 46211 and 20265 in the presence of doxycycline respectively (Figure 3B and data not shown).

### The *TetR* protein efficiently localize to the nucleus

**Figure 4:**
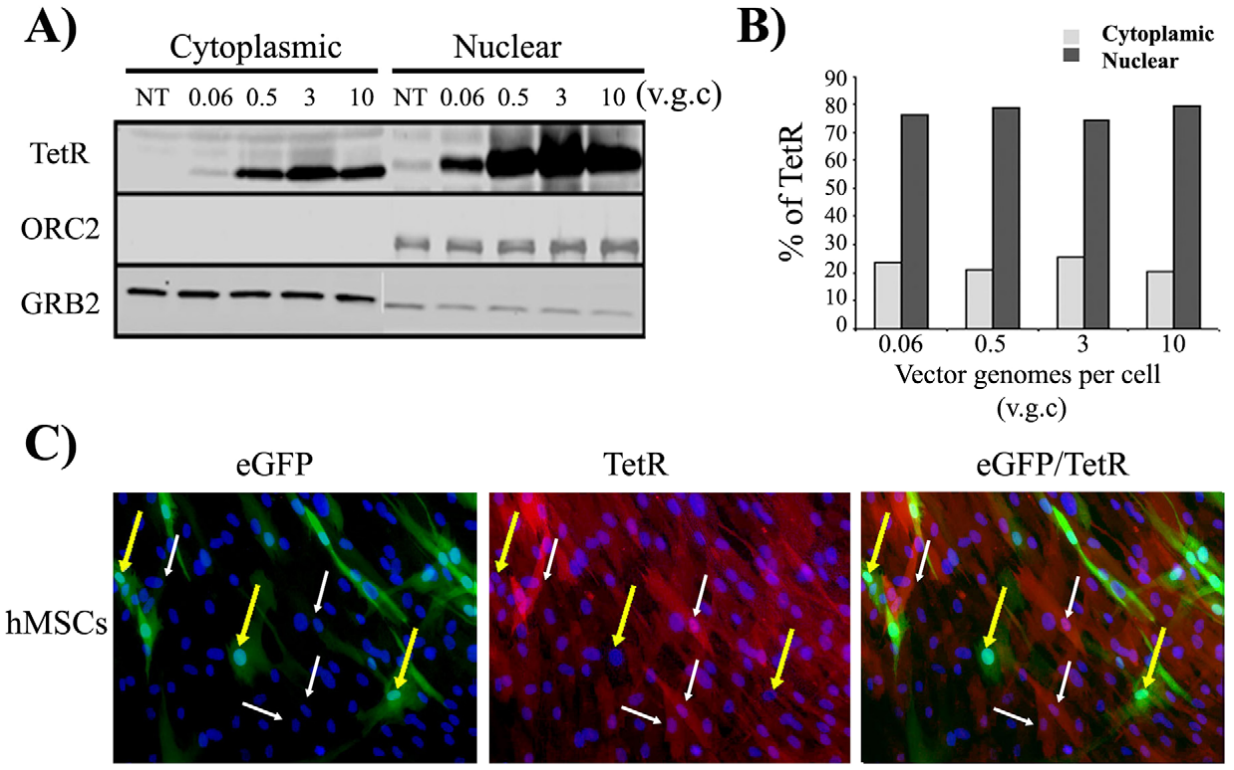
Nuclear localization of *TetR* repressor in CEST-transduced cells correlates with complete repression of CMV promoter. **A)**. Quantitative Western blot analysis showing *TetR* expression in cytoplasmic and nuclear fraction of 293 T cells transduced with increasing CEST MOIs. Cell fractionation was carried out as indicated in M&M. ORC2 and GRB2 were used as markers for the nuclear and cytoplasmic fraction respectively, and as a loading control. **B)**. Graph showing percentage of *TetR* repressor that is present in the cytoplasmic (gray bars) and nuclear (black bars) fractions as determined by densitometry analysis of the Western blot (A). Note that the *TetR* protein is mainly localized within the nucleus, independently of the *TetR* concentration. **C)**. Immunofluorescence staining of *TetR* in CEST transduced hMSCs (2 v.g.c.). Cells were fixed and incubated with monoclonal IgG1 against *TetR* amino acid 84-98 and incubated with DAPI (marking nuclei in blue) as indicated in M&M. Left panel shows a picture that has been manually enhanced to visualize minimally expressed eGFP. Middle and right pictures show *TetR* expression and *eGFP/TetR* merge images respectively. White arrows indicate cells which are completely negative for eGFP expression. Note the purple colour of these cells (middle and right panels) as consequence of the co-localization of DAPI (Blue) and red (*TetR*). Yellow arrows indicates cell expressing low levels of *TetR* repressor and, therefore expressing eGFP (left and right panels). The nuclei of these cells are intense blue (middle panel) indicating the absence of *TetR* expression. v.g.c.; vector genomes per cell. NT; not transduced.

Theoretically, if the amount of *TetR* repressor expressed by one copy of the CEST vector is not enough to control one TetO operon (as demonstrated by the absence of modulation at low MOI), cells containing multiple copies of the CEST should have even higher leakiness. One possible explanation could be an inefficient nuclear transport of the *TetR* repressor and the subsequent requirement of hight *TetR* concentrations in the cytoplasm to get *TetR* into the nuclei. To test this hypothesis, we analysed *TetR* content in the cytoplasmic and nuclear fractions of 293 T cells transduced at different MOIs. The results showed that up to 80% of the *TetR* localize in the nuclei even at low MOI (Figure 4A; 0.06 v.g.c.). In addition, the percentage of *TetR* repressor present in the nuclear versus the cytoplasmic fractions was very similar independently of the concentration of the *TetR* (Figure 4B). These data indicate that *TetR* is transported to the nuclei quite efficiently independently of the total *TetR* cell content.

We further analyzed the level of correlation between *eGFP* expression and *TetR* concentration and/or nuclear localization. We performed immunostaining of CEST-transduced hMSCs containing an average of 2 v.g.c. (Figure 4 C). These bulk CEST-hMSCs contained highly-responsive cells (as observed in Figure 3C middle graphs and data not shown) but they also contained a percentage of cells that were inadequately regulated (cells that are eGFP^+^ in the absence of doxycycline). Due to the low eGFP expression levels of some cells, the pictures taken with the fluorescence microscopy were further enhanced to visualize any residual *eGFP* expression. The aim was to correlate eGFP expression levels (Figure 4C left panels) with *TetR* expression (Figure 4C middle panels). Cells expressing higher amounts of *TetR* (white arrows) were completely negative for *eGFP* expression. In these cells, a significant part of the *TetR* was located in the nuclei as indicated by the purple colour of their nucleus as result of the co-localization of red (*TetR*) and blue (DAPI) colours (Figure 4C white arrows, middle panel). On the contrary, cells expressing *eGFP* were negative for *TetR* expression (Figure 4C, yellow arrows, middle panel) as demonstrated by the absence of colocalization of the green and red colours (Figure 4C right panels).

### Doxycycline-responsive primary cell lines maintain the main properties of parental cells

**Figure 5:**
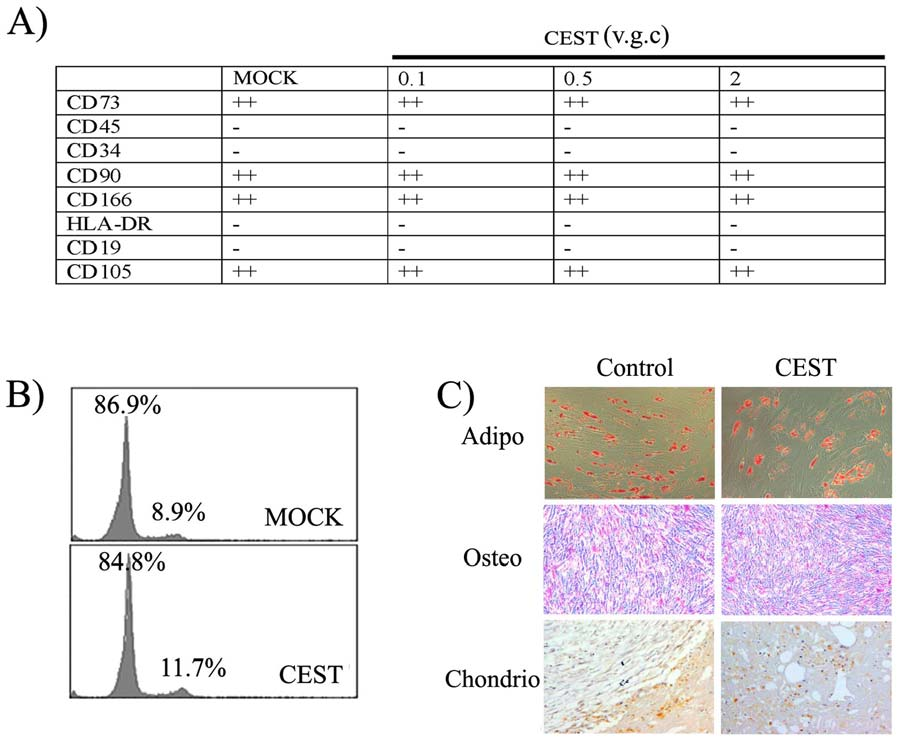
Doxycycline-responsive human mesenchymal stem cells (hMSCs) maintain the main properties of parental hMSCs. **A**) Different doxycycline-responsive hMSCs were generated with increasing MOIs of the CEST vector to obtain an average of 0.1, 0.5, and 2 v.g.c. (indicated on the top). Expression of the different surface markers were analyzed by flow cytometry and compared to the expression by a parental (MOCK-transduced) hMSCs. The hMSCs containing 2 v.g.c. of the CEST vector was further analyzed to test the influence of vector expression on cell cycle status **B**) and on differentiation potential toward adipogenesis (top panels), osteogenesis (middle) and chondriogenesis (bottom panles) **C**). No significant differences were observed between the parental and the CEST-transduced hMSCs.

MSCs are an important target for cell-gene therapy applications. Their role in regeneration, immunomodulation as well as their migratory capabilities to inflammatory locations makes them an attractive target for gene manipulation. The development of an efficient doxycycline-responsive gene transfer system to achieve high levels of transgene expression in MSCs (without affecting its phenotype) is of special interest for the field. We therefore studied potential changes on doxycycline-responsive hMSCs compared with parental hMSCs. We compared control hMSCs (MOCK) with hMSCs containing increasing amounts of integrated CEST vectors. We study potential variations in the expression of positive (CD73, CD90, CD105, CD166) and negative (CD19, CD34, CD45, and HLA-DR) surface markers. We also studied potential alteration in proliferation and/or apoptosis in hMSCs containing 2 v.g.c. by analyzing cell cycle status with Propidium Iodide. We did not detect any changes on neither the expression of the main surface markers (Figure 5A) nor in the cell cycle status (Figure 5B).

Another important characteristic of MSCs is their ability to differentiate toward different cell types. We therefore studied whether vector transduction affect the ability of MSCs to differentiate to adipocytes, osteocytes and chondriocytes. As can be observed in Figure 5C, neither CE (expressing only eGFP) nor CEST (expressing eGFP and *TetR*) transduction of MSCs had any detectable impact on the differentiation potential of MSCs.

## Discussion

In the present work we have developed a *TetR*-based dual and all-in-one lentiviral system that efficiently generate doxycycline-responsive cell lines without the requirements of cloning and/or antibiotic selection. Most of the doxycycline-responsive systems are based on the TetR-VP16 chymeras (tTA and rtTA) [2], [3], [4], [5], [6]. In these systems, the promoter is only active when the tTA or the rtTA transactivators bind the regulated promoter. The expression of the VP16 transactivators in the regulated cells can have several undesired consequences such as alteration of the promoter natural activity, activation of cellular genes and toxicity [7], [8], [9], [10], [11]. We based our studies in a new doxycycline-regulated system based on the original *TetR* repressor developed in 1998 by Yao et al. [12] as an alternative to the *TetR-VP16* chimeras (*tTA* and *rtTA*).

We first developed two lentiviral vectors; one driving the expression of the original *TetR* repressor through the SFFV promoter (STetR) and a second one harbouring the CMVTetO cassette expressing eGFP. We used the SFFV promoter to drive *TetR* expression for its well-known characteristic as a strong promoter in several cell types, including important targets for gene therapy [25], [27], [28]. The STetR lentiviral vector was used to generate different cell lines expressing variable levels of *TetR* repressor by increasing the MOI. These cell lines allowed us to study the minimal requirement of *TetR* concentration needed for the regulation of the CMVTetO operon. Previous studies are somehow contradictory regarding the *TetR* repressor concentrations required to achieve tetracycline regulation. While Yao et al. propose that *TetR* concentration must be kept high to obtain good tetracycline responsiveness[12], [19], other authors argue that minimal amounts of *TetR* could be sufficient to shut off the TetO-regulatable CMV promoter[20]. The fact that most (if not all) recent publications using the *TetR* repressor system required cloning selection or antibiotic selection [15], [16], [17], [18] to generate regulatable cell lines favour the theory of Yao et al. In this direction we have done a systematic study demonstrating that in order to achieve good inducibility and low leakiness, the cells must contain al least 2 copies of the StetR vector integrated. For maximal responsiveness, the cells must have over 4 vectors genomes of STetR per cell that, as shown by Western blot analysis (Figure 4B), produce very high concentrations of *TetR* repressor.

The different TetR-293T cell lines were further used to study if the TetO concentrations are a limiting factor as occur for the rtTA system. Transactivator-containing repressors (rtTA) are quite toxic for most of the cells and must be kept at a low concentration. Therefore, the concentration of TetO binding sites must be kept low to equal low repressor concentrations[7], [11], [26]. Contrary to rtTA-based systems, *TetR* concentrations inside the regulated cells were able to modulate high amounts of TetO sequences (present in the CTetOE vector). Since the leaking was similar in cells harbouring increasing copies of the CTetOE (in cells harbouring over 4 StetR v.g.c.) and the expression levels in the presence of doxycycline augmented proportionally to the amount of CTetOE v.g.c, the final fold induction index was always better for cells containing multiple copies of the CTetOE vector. This vector module can therefore be used to generate regulatable cell lines expressing increasing amount of the transgene simply by increasing the MOI of the CTetOE vector. Each of the different cell lines generated can additionally be modulated by adjusting doxycycline concentrations.

Although a two vector system has important applications both in basic research and cell-gene therapy therapeutic approaches, the availability of all-in-one vector harbouring all the necessary elements to regulate a transgene is highly desirable for several applications including *in vivo* application and the generation of regulatable human primary cells. All-in-one vectors have the additional advantage of minimising the amount of integrative vectors required for transgene modulation and therefore reduce the potential alteration of regulatable cell lines We have therefore generated an all-in-one *TetR*-based lentiviral vector (CEST) module for the generation of immortalized and primary human doxycycline-responsive cell lines. The CEST vector produce over 10.000.000 tu/ml and one transduction with concentrated vector were able to generate highly-responsive immortalized (293T) and primary (hMSCs and HEF) cells.

The necessity of multiple copies of the CEST vector to regulate the transgene confirm the requirement of high levels of the *TetR* repressor and, at the same time, also confirms that once this levels are achieved, they can modulate high amounts of CMVTeO expression cassettes. Indeed, in CEST-transduced cells, the cells expressing higher levels of *TetR* repressor have also higher levels of CMVTetO to regulate. Still the leakiness drop and the fold induction increased proportionally to CEST vector genomes per cell (Figure 3). These data support the hypothesis that high concentrations of the *TetR* repressor is the main factor required to get good doxycycline regulation. In fact increasing the numbers of TetO targets up to 20 times with the CEST do not preclude significantly *TetR* repression but it does augment transgene expression and therefore increase total fold induction. This property allowed us to control transgene expression not only by adjusting doxycycline concentrations but also by controlling the MOI used for transduction of the target cells. We also showed that the system is highly responsive to low doses of doxycycline with 1 ng/ml being sufficient to reach peak expression in cells containing up to 4 v.g.c. However in cell lines with very high amounts of CEST vector integrated (20 v.g.c.), the maximum inducibility was achieved with 10 ng/ml of doxycycline. The fact that high MOIs of the CEST showed lower leakiness than low MOIs was surprising and probably reflects some way of *TetR* cooperation to achieve promoter shut down. We have shown that in 293 T and MSCs, the *TetR* repressor is transported inside the nucleus quite efficiently (up to 80% of the total cell content) and independently of the *TetR* concentration eliminating the possibility of *TetR* cooperation to reach the nucleus as a factor involved in this phenomenon. Therefore, we favour the hypothesis of a requirement of high concentration of *TetR* to cooperate for the formation of *TetR* homodimers required for promoter shut down. At low MOIs, the *TetR* concentration does not reach the minimum required and are therefore unable to modulate transgene expression.

An important factor related to the use of the original *TetR* repressor is the absence of toxic effects. We analyzed the potential alteration of *TetR* overexpression in hMSC, an important target for cell-gene therapy applications because of its role in regeneration and immunomodulation. We did not found any alteration on the phenotype, cell cycle status or differentiation potential on hMSCs that were transduced at high MOI with the CEST vector and that were highly-responsive to doxycycline. However, the fold induction index of the CEST-transduced MSCs was slightly lower than CEST-transduced 293 T and HEF cells, probably due to the differences in CEST vector genome per cell achieved (293 T and HEF cells are 8–4 times more permissive than hMSCs).

Although both, the dual (StetR and CTetOE) and the all-in-one-based (CEST) vectors described in this manuscript, are highly efficient for the development of doxycycline-regulated cell lines, the requirement of multiple v.g.c. to achieve regulation is an important drawback of the system. Indeed multiple integrations are more likely to alter physiology of the doxycycline-regulated cells and the system work poorly in highly restrictive cells such as CD34+ cells (data not shown). The ideal vector should allow complete CMVTetO promoter inhibition with only one vector integration. We are at the moment trying to improve the system by comparing various nuclear localization signals on the *TetR* repressor, by insulating the all-in one vector and by using alternate vector modules to increase *TetR* production. We have evidences that different cell lines behave differently in term of nuclear localization of the original *TetR* and that these differences correlate with differences in doxycycline regulation (our unpublished data). Therefore, the use of nuclear localization signal together with further improvements in the vector should improve not only the amount of cell lines that can be modulated but also will open the window to its use for *in vivo* applications.

## Materials and Methods

### Cells and reagents

293 T cells (Chantret *et al.* 1994) were maintained in Dulbecco's Modified Eagle's Medium (DMEM, Invitrogen) supplemented with 10% Fetal Bovine Serum (FBS, Invitrogen), 1% essential amino-acids and antibiotics. Human mesenchymal stem cells (hMSCs) were obtained from Inbiobank (www.inbiobank.org; San Sebastian, Spain), and were cultured in Advanced-DMEM (Gibco) plus 10% FBS. When cell cultures achieved over 85% of density, adherent cells were trypsinized, washed in PBS and re-plated at a concentration of 5×10^3^ cells/cm^2^. HFFs were purchased from ATCC (SCD-1112SK). During routine maintenance, HFFs were grown in IMDM, plus 10% FCS and 2 mM L-glutamine and split in the ratio 1∶2 when they reached 80-90% confluence. K562 were obtained from the ATCC (CCL-243) and maintained in RPMI media (Invitrogen), supplemented with 10% Fetal Bovine Serum (Invitrogen).

### Plasmids construction

#### Dual system

StetR and CTetOE. The StetR vector plasmid was obtained by replacing *EGFP* cDNA from the pHRSIN-CSEW [25] plasmid (using BamHI and NotI excision) with a *TetR* cDNA obtained by PCR using pcDNA6TR (Invitrogene) as a template and the BamH1-TetR Fw (5′ GGATCCATGTCTAGATTAGATAAAAG) and Not-TetR reverse (5′ GCGGCCGCTTAATAAGATCTGAATTCCCGGG). Primers to include the BamHI NotI sites at both ends. To construct the CTetOE vector plasmid, we used pHRSIN-CSEW vector as backbone, excising the SFFV promoter using EcoRI/BamHI restriction enzymes. A PCR fragment containing CMVTetO cassette was obtained by PCR using the EcoR1 forward (CCGGAATTCGTTGACATTGATTATTGACTA) and BamH1 reverse (CGCGGATCCCGGAAGATGGATCGGTCC) primers and the pcDNA4/TO plasmid (Invitrogene) as template.

#### All-in-one CEST lentiviral vector

The CEST vector plasmid harbouring the CMV-TetO regulatable cassette driving the expression of *GFP* as well as the spleen focus-forming virus promoter driving the expression of the *TetR* repressor gene was constructed by cloning the SFFV-TetR PstI fragment from the STetR vector into the unique Pst1 restriction site of the CTetOE vector.

### Vector production and titration

The HIV packing plasmid (pCMVDR 8.9) and VSG-G plasmid (pMD.G) are described elsewhere (Naldini et al., 1996; Zufferey et al., 1998). Vector production was performed as described previously (Toscano et al., 2004). Briefly, 293 T cells, were plated, and the vector, packaging, and envelope plasmids (plasmid proportion 3∶2∶1) were resuspended in 1.5 ml of Opti-MEM (GIBCO) mixed with 60 µl of Lipofectamine 2000 (Invitrogen, Carlsbad, CA) and diluted in 1.5 ml of Opti-MEM (GIBCO). The mixture was added to the 293 T cells, which were incubated for 6–8 h, washed and cultured for an additional 48 h. Viral supernants were collected and filtered through a 0.45 µm (pore size) filter (Nalgene, Rochester, NY) aliquoted and immediately frozen at −80°C. For the titration of vectors, transduced cells were lysed and DNA extracted after 7–10 days, and vector copy number per genome (v.c.g) was determined using quantitative PCR as described below.

### DNA extraction and quantitative real-time PCR

Genomic DNA was isolated by adding 1 ml per 10^6^ cells of SNET extraction buffer (20 mM Tris-HCl [pH 8], 5 mM EDTA [pH 8], 400 mM NaCl, 1% SDS) containing proteinase K (100 mg/ml; Sigma- Aldrich). DNA samples were incubated at 55°C for 2 hr, proteinase K was inactivated by incubating at 98°C for 10 min, and RNase (1 mg/ml) was finally added for 30 min at 37°C. Proteins were extracted twice with phenol–chloroform, and DNA was then precipitated and its concentration determined by spectrophotometry. Quantitative real-time PCR were performed with an Mx3005P system (Agilent). The real time PCRs were performed using the QuantiTect™ SYBR Green PCR Kit (from Qiagen). To quantitate CEST lentiviral integration we used primers for the WPRE sequence; WPRE-F: 5′- CACCACCTGTCAGCTCCTTT and WPRE-R: 5′- ACAACACCACGGAATTGTCA. For the STetR vector we used primers for *TetR* sequence, TetR–F: 5′ GGGATCCTAGTGATTATGTCT and TetR-R: 5′ TTACGGGTTGTTAAACCTTC and for the CTetOE vector we used primers for the eGFP sequence, GFP-F: 5′GTTCATCTGCACCACCGGCAAG and GFP-R TTCGGGCATGGCGGACTTGA. The parameters for the PCR were 1× (95°C, 2 min); 40×(95°C, 15 sec / 55°C, 30 sec / 72°C, 30 sec); 1×(72°C 2 min). We calculated the vector copy number per genome by interpolation in the standard curve made with 10-fold increasing amounts of plasmid DNA (CTetOE)) from 10^3^ to 10^7^ v.g.c. and by starting with 0.6 µg genomic DNA (about 100.000 genomes).

### Cell extraction and Western blotting

Cytosolic and nuclear fractions of transduced and controls cells were obtained using the Qproteome nuclear protein Kit (Qiagen) following the manufacturer's instruction. Proteins were resolved by sodium dodecyl sulfate– polyacrylamide gel electrophoresis (SDS–PAGE; 10% polyacrylamide gels, reducing conditions), and electrotransferred to Hybond-P polyvinylidene difluoride (PVDF) membranes (GE Healthcare Life Sciences, Buckinghamshire, UK). Membranes were blocked with 5% nonfat milk and probed for 1 hr at room temperature with rabbit anti-GRB2 (BD pharmingen; no 559266), rabbit anti-ORC (BD Pharmigen, no 559166) and mouse anti-Tet-repressor (Mobitec; no TET02). Combination of IRDye 680LT Goat anti-Rabbit IgG (Licors: no 26-68021) and IRDye 800CW Goat anti-Mouse IgG (Licors: no 926-32210) were use at 1:10.000 dilutions to analyzed *TetR* protein in combination with either ORC or GRB2. After washing the membranes, detection and quantification of protein were performed using Odyssey Image Scanner System (Licor Biosciences, Cambridge, UK) using IRdye-conjugated secondary antibodies (Licor) and the Odyssey quantification software.

### Inmunostaining

For immunofluorescence analysis of cultured cells, cells were fixed in 4% paraformaldehyde-PBS for 20 min, permeabilized with 0.1–1% Triton X-100-PBS for 15 min, and blocked with 5% PBS for 45 min at room temperature (RT). Fixed cells were incubated with 2 µg/ml anti-Tet-repressor (Mobitec; TET02) and then with a secondary Texas-red-conjugated anti-mouse IgG (Becton Dickinson (BD)). Stained cells were then mounted in Vectashield mounting medium with DAPI (H-1500, vector laboratories) to stain the nuclei and examined using an Olympus AX60 fluorescence microscope.

### Phenotype of hMSCs

MSCs were collected, washed and pre-incubated in a PBS-blocking solution containing 3% of fetal bovine serum (FBS) and 0,2% sodium azide for 15 minutes at 4°C. The following antibodies were used to fully characterize expression pattern of transduced and untransduced MSCs: anti human CD90-FITC, CD73-PE, CD105-FITC, CD166-PE, CD106-PE, CD45-PerCP, CD34-APC, HLA-DR- PerCP, CD19-APC all from Becton Dickinson (BD). Antibodies were diluted in PBS 0,3% of FBS and 0,02% sodium azide. Cells were incubated with 100 µl of the different antibodies (1/100), for 1 h at 4°C and agitation and washed in PBS 0,3% of FBS and 0,02% sodium azide followed by a final wash in PBS alone. Cells were analyzed on FACS Canto Flow Cytometer.

### Adipocyte, Osteocyte and chondriocyte differentiation

Differentiation potential of hMSCs was studied by incubating the different cell lines in specific differentiation inductive media (Lonza, Basel, Switzerland). For adipogenic differentiation, cells were cultured in Adipogenic MSCs differentiation BulletKit (Lonza, Basel, Switzerland) and stained with Oil Red O (Amresco, Solon, OH). For osteogenic differentiation, cells were cultured in Osteogenic MSCs differentiation BulletKit (Lonza, Basel, Switzerland) and stained with Alizarin Red S (Sigma). For chondrogenic differentiation, cells were cultured in chondrogenic MSCs differentiation BulletKit (Lonza, Basel, Switzerland), differentiated cells were include in paraffin block, and chondrocyte-like cells were inmunodetected with Anti-S100 (*Dako*, polyclonal rabbit *anti*-*S100*, code Z0311).

### Fold induction index and leakiness determination

Transduced cells incubated in the presence or absence of different concentrations of doxycycline were analyzed by flow cytometry to determine the percentage of eGFP positive cells and the Mean fluorescence intensity (MFI) of either, the eGFP+ population or the entire population.

The *Fold induction index* was estimated as arbitrary units obtained by following formula:

[% eGFP^+^ (+Dox) / %eGFP^+^(-Dox)] x [MFI eGFP^+^ cells (+Dox) / MFI eGFP^+^ cells (-Dox)]

*Leakiness* of the system was determined by using the following formula:

[% eGFP^+^ (-Dox) / %eGFP^+^(+Dox)] x MFI eGFP^+^ cells (-Dox)

### Cell cycle analysis

Cell cycle assays were performed as previously described[29]. Briefly, the trypsinized cells were fixed in 70% ethanol, washed with PBS, and then incubated with Propidium Iodide (20 mg/ml) in PBS containing RNase A for 30 min at 37^a^C. After washing, cells were analyzed by flow cytometry (FACS Canto, Becton Dickenson).
